# Hidden Historical Habitat-Linked Population Divergence and Contemporary Gene Flow of a Deep-Sea Patellogastropod Limpet

**DOI:** 10.1093/molbev/msab278

**Published:** 2021-09-17

**Authors:** Ting Xu, Yan Wang, Jin Sun, Chong Chen, Hiromi Kayama Watanabe, Junlin Chen, Pei-Yuan Qian, Jian-Wen Qiu

**Affiliations:** 1 Department of Ocean Science and Hong Kong Branch of the Southern Marine Science and Engineering Guangdong Laboratory (Guangzhou), The Hong Kong University of Science and Technology, Hong Kong, China; 2 Southern Marine Science and Engineering Guangdong Laboratory (Guangzhou), Guangzhou, China; 3 Department of Biology and Hong Kong Branch of the Southern Marine Science and Engineering Guangdong Laboratory (Guangzhou), Hong Kong Baptist University, Hong Kong, China; 4 Institute of Evolution and Marine Biodiversity, Ocean University of China, Qingdao, China; 5 X-STAR, Japan Agency for Marine-Earth Science and Technology (JAMSTEC), Yokosuka, Kanagawa, Japan

**Keywords:** hydrothermal vent, hydrocarbon seep, patellogastropod, demographic history, population connectivity, physical ocean modeling

## Abstract

Hydrothermal vents and hydrocarbon seeps in the deep ocean are rare oases fueled by chemosynthesis. Biological communities inhabiting these ecosystems are often distributed in widely separated habitats, raising intriguing questions on how these organisms achieve connectivity and whether habitat types facilitate intraspecific divergence. The deep-sea patellogastropod limpet *Bathyacmaea nipponica* that colonizes both vents and seeps across ∼2,400 km in the Northwest Pacific is a feasible model to answer these questions. We analyzed 123 individuals from four vents and three seeps using a comprehensive method incorporating population genomics and physical ocean modeling. Genome survey sequencing and genotyping-by-sequencing resulted in 9,838 single-nucleotide polymorphisms for population genomic analyses. Genetic divergence and demographic analyses revealed four habitat-linked (i.e., three seep and one vent) genetic groups, with the vent genetic group established via the opportunistic invasion of a few limpet larvae from a nearby seep genetic group. TreeMix analysis uncovered three historical seep-to-vent migration events. ADMIXTURE and divMigrate analyses elucidated weak contemporary gene flow from a seep genetic group to the vent genetic group. Physical ocean modeling underlined the potential roles of seafloor topography and ocean currents in shaping the genetic connectivity, contemporary migration, and local hybridization of these deep-sea limpets. Our study highlighted the power of integrating genomic and oceanographic approaches in deciphering the demography and diversification of deep-sea organisms. Given the increasing anthropogenic activities (e.g., mining and gas hydrate extraction) affecting the deep ocean, our results have implications for the conservation of deep-sea biodiversity and establishment of marine protected areas.

## Introduction

The discoveries of deep-sea hydrothermal vents in the late 1970s and hydrocarbon seeps in the early 1980s have significantly changed our understanding of how life has evolved on Earth. Unlike shallow-water ecosystems that are mainly driven by photosynthesis, deep-sea vent and seep ecosystems distributed in tectonically active areas and along continental margins are primarily supported by chemosynthesis, with many species strictly endemic to either vent fields or seep areas (Van Dover et al. 2002). Nevertheless, a number of species are capable of thriving in both habitats (Wolff 2005; Watanabe et al. 2010), raising intriguing questions on how their populations achieve connectivity between widely separated habitats and whether differences in habitat types facilitate intraspecific divergence. Studying species that inhabit both vents and seeps may allow us to address these issues by shedding light on their historical colonization, migration dynamics, and adaptive evolution (Vrijenhoek 2010; Baco et al. 2016). Such information will in turn contribute to the evaluation of resilience of vent- and seep-associated macrofauna after anthropogenic disturbances, management of deep-sea resources, and designation of marine-protected areas (Baco et al. 2016; Breusing et al. 2016; Levin et al. 2020).

With over 90 hydrothermal vents and 70 hydrocarbon seeps discovered, and over 20% macrobenthic species shared across these two types of habitats, the Northwest Pacific has been recognized as an evolutionary hotspot for chemosynthesis-based biota (Watanabe et al. 2010; Feng et al. 2018; Beaulieu and Szafrański 2020). Previous population genetic and genomic studies of macrobenthos dwelling in both hydrothermal vents and hydrocarbon seeps in this region have mainly focused on the bathymodioline mussel *Gigantidas platifrons* and the galatheoid squat lobster *Shinkaia crosnieri*. Bathymodioline mussels, at least the species for which the dispersal mode has been studied, produce planktotrophic larvae that can disperse a long distance in the upper ocean currents (Arellano et al. 2014; Laming et al. 2018). A population genomic study based on genome-wide single-nucleotide polymorphisms (SNPs) revealed two semi-isolated lineages of *G*. *platifrons* in the Northwest Pacific, with one mainly inhabiting the Jiaolong Ridge seep in the marginal South China Sea and the other distributed in vent fields in the Okinawa Trough and the Off Hatsushima seep in the Sagami Bay (Xu et al. 2018). Moreover, a minor genetic subdivision was unveiled between its local populations in the southern Okinawa Trough and those in the middle Okinawa Trough and the Sagami Bay. These results reinforced the Luzon Strait and the intra-trough grabens between the southern and the middle Okinawa Trough as potential geographic barriers that may have accelerated the local adaption and genetic differentiation of *G*. *platifrons* (Xu et al. 2018). Unlike bathymodioline mussels, the galatheoid squat lobster *S*. *crosnieri* produces lecithotrophic larvae that are expected to mainly drift in the middle to the deeper water layers (Miyake et al. 2010; Xiao et al. 2020). Population genetic and genomic studies based on gene markers, genome-wide SNPs, and transcriptome-wide SNPs consistently disclosed a clear genetic division between its seep population in the South China Sea and vent populations in the Okinawa Trough, also highlighting the barrier effect of the Luzon Strait and the environmental adaptabilities of *S. crosnieri* to the local conditions (Shen et al. 2016; Yang et al. 2016; Cheng et al. 2020; Xiao et al. 2020) . These studies have broadened our knowledge of the population connectivity and genetic divergence of two representative macrobenthos in vent and seep ecosystems. Nonetheless, how other vent and seep dominant macrobenthos achieve connectivity amongst geographically and geochemically distinct habitats, and whether they display a habitat-linked demographic history have not been critically examined.

**Fig. 1.**
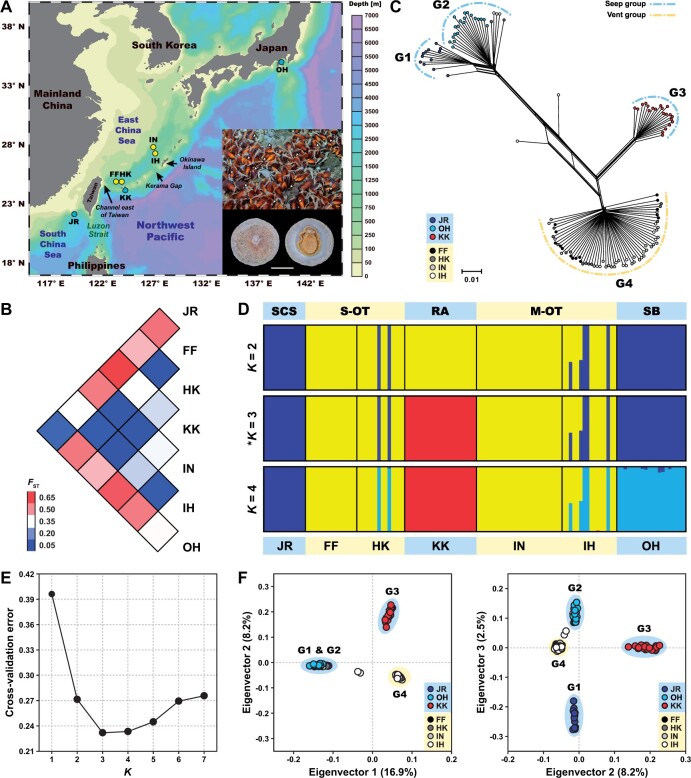
Population divergence of *Bathyacmaea nipponica*. (*A*) Sampling vents (yellow dots) and seeps (blue dots). The map was created using Ocean Data View (ODV) v.5.0 (https://odv.awi.de). Inset: a representative photograph of the Futagoyama Field vent (upper) showing *B*. *nipponica* (indicated by white arrows) on shells of the bathymodioline mussel *Gigantidas platifrons*, along with the dorsal and ventral views of a *B*. *nipponica* specimen (lower). Scale bar = 1 cm. (*B*) Pairwise *F*_ST_ values between the local population pairs indicating population genetic differentiation estimated using Arlequin. (*C*) A phylogenetic network revealing four habitat-linked genetic groups (i.e., G1–G4) reconstructed by SplitsTree. (*D*) Population structure and individual ancestry detected via ADMIXTURE under two to four predefined genetic groups, with the optimal number (i.e., *K = *3) indicated with an asterisk. (*E*) Cross-validation (CV) error calculated by ADMIXTURE was the smallest at *K *=* *3, however, the value at *K *=* *4 was very close to that at *K *=* *3. (*F*) PCA revealing a habitat-linked genetic divergence pattern amongst *B*. *nipponica* individuals by SNPRelate. In (*C*), (*D*), and (*F*), vent and seep local populations or genetic groups are shaded in yellow and blue, respectively. Abbreviations for localities of local populations or genetic groups: JR, Jiaolong Ridge seep; FF, Futagoyama Field vent; HK, Hatoma Knoll vent; KK, Kuroshima Knoll seep; IN, Iheya North Field vent; IH, Izena Hole Field vent; OH, Off Hatsushima seep; SCS, South China Sea; S-OT, southern Okinawa Trough; RA, Ryukyu Arc; M-OT, middle Okinawa Trough; SB, Sagami Bay.

The patellogastropod limpet *Bathyacmaea nipponica* is amongst the most widely distributed macrobenthos across chemosynthesis-based ecosystems in the Northwest Pacific, and can therefore serve as a model organism to fill such knowledge gaps. *Bathyacmaea nipponica* mainly grazes bacterial film on various hard substrates for nutrition, and this species has been considered to produce pelagic-dispersing lecithotrophic larvae as is typical in patellogastropod limpets (Chen et al. 2019; Ponder et al. 2020). To date, *B*. *nipponica* has been found in vent fields in the Okinawa Trough and hydrocarbon seeps in the South China Sea, the Ryukyu Arc, and the Sagami Bay (fig. 1*A*), with depths ranging between 625 to 1,684 m (http://www.godac.jamstec.go.jp/bio-sample/index_e.html, last accessed January 15, 2021) (Watanabe et al. 2010; Zhao et al. 2020). Here, we applied a comprehensive approach incorporating population genomics and physical ocean modeling to examine the demographic history and population connectivity of *B*. *nipponica*. Through this study, we aimed to 1) understand the habitat colonization and evolutionary divergence of its vent and seep populations; 2) test whether these populations exhibit a habitat-linked genetic differentiation; and 3) investigate the migration dynamics and gene flow of *B*. *nipponica* under the intricate interactions amongst local habitats, seafloor topography, and ocean currents.

## Results

### Assembly of *B*. *nipponica* Survey Genome and Detection of Genome-Wide SNPs

The Illumina whole-genome sequencing generated 111.2 Gb of raw reads. After removing low-quality reads, a total of 102.9 Gb reads were retained for genome assembly using Platanus, which produced 2,733,936 scaffolds with a total size of 950.7 Mb and an N50 of ∼2.3 kb (supplementary table S1, Supplementary Material online). BUSCO analysis indicated that this assembly contained 88.4% of the conserved metazoan genes (i.e., 59.2% complete [58.8% single copy; 0.4% duplicated] + 29.2% fragmented BUSCOs), providing a feasible reference genome for downstream population genomic analyses. Sequencing of 123 genotyping-by-sequencing libraries produced an average of 4.7 million high-quality reads for each individual (supplementary table S2, Supplementary Material online). After genotyping and strict filtering, a total of 9,838 SNPs were identified from 2,591 scaffolds (supplementary table S3, Supplementary Material online).

### Genetic Differentiation

Genetic differentiation measured by pairwise *F*_ST_ between all the local population pairs ranged between 0.0054 and 0.6137 (fig. 1*B* and supplementary table S4, Supplementary Material online). In particular, the pairwise *F*_ST_ values were high between the Jiaolong Ridge and the Off Hatsushima seep populations and all the other local populations (range: 0.3415–0.6137), intermediate between the Kuroshima Knoll seep population and the Okinawa Trough vent populations (range: 0.2777–0.3431), and very low between the vent local population pairs in the Okinawa Trough (range: 0.0054–0.0475).

### Phylogenetic Network

The phylogenetic network showed four habitat-linked genetic groups, including three seeps and one vent genetic groups (fig. 1*C*). Specifically, the first seep genetic group (G1) comprised all individuals from the Jiaolong Ridge seep in the South China Sea. The second seep genetic group (G2) included all individuals from the Off Hatsushima seep in the Sagami Bay, together with two individuals from the Hatoma Knoll vent in the southern Okinawa Trough and three individuals from the Izena Hole Field vent in the middle Okinawa Trough. The third seep genetic group (G3) contained all individuals from the Kuroshima Knoll seep in the Ryukyu Arc. The fourth vent genetic group (G4) consisted of the rest individuals from the four vent fields in the Okinawa Trough.

### Population Structure and Individual Assignment

ADMIXTURE analysis revealed a habitat-linked genetic structure (fig. 1*D* and *E*) that was in line with the phylogenetic network (fig. 1*C*). At *K *=* *2, all individuals from the Jiaolong Ridge and the Off Hatsushima seeps, together with two individuals from the Hatoma Knoll vent and three individuals from the Izena Hole Field vent in the Okinawa Trough, formed one genetic group. Most of the other individuals from the Okinawa Trough vents along with all individuals from the Kuroshima Knoll seep formed the other genetic group. In addition, two individuals from the Izena Hole Field vent were detected to exhibit genetic architecture of both genetic groups. At *K *=* *3, all individuals from the Kuroshima Knoll seep were segregated from those inhabiting the Okinawa Trough vents to form the third genetic group. At *K *=* *4, all individuals from the Jiaolong Ridge seep and the Off Hatsushima seep were further separated to form two distinct genetic groups. Moreover, two individuals from the Hatoma Knoll vent and three individuals from the Izena Hole Field vent in the Okinawa Trough were found to be migrants from the Off Hatsushima seep, and two individuals from the Izena Hole Field vent were detected to be hybrid descendants between the Okinawa Trough vent and the Off Hatsushima seep genetic groups.

Principal components analysis (PCA) provided further evidence to support a habitat-linked population divergence (fig. 1*F*). In particular, the population divergence pattern displayed along the first two eigenvectors (i.e., G1 and G2; G3; G4) agreed with the ADMIXTURE results at *K *=* *3 (fig. 1*D*). Additionally, the G1 and G2 genetic group was separated into two distinct genetic groups G1 and G2 along the third eigenvector, with G1 containing all individuals from the Jiaolong Ridge seep, and G2 including all individuals from the Off Hatsushima seep along with two individuals from the Hatoma Knoll vent and three individuals from the Izena Hole Field vent in the Okinawa Trough. These results were consistent with the reconstructed phylogenetic network (fig. 1*C*) and the genetic structure revealed by ADMIXTURE (*K *=* *4; fig. 1*D*).

### Population Genetic Statistics

The Okinawa Trough vent genetic group had the greatest number of polymorphic nucleotide sites (6,554; 66.6%) and private SNPs (2,886; 29.3%), as well as the highest values of the observed heterozygosity (*H*_obs_), nucleotide diversity (*π*), and inbreeding coefficient (*F*_IS_), compared with the other three seep genetic groups (table 1 and supplementary table S5, Supplementary Material online).

**Table 1.** Summary Genetic Statistics for the Four Genetic Groups of *Bathyacmaea nipponica*.

Habitat	Genetic Group	*N*	Variant	Poly	Poly (%)	Private	Private (%)	*H*_exp_	*H*_obs_	*π*	*F*_IS_
Seep	JR	12	9,705	2,676	27.6	55	0.6	0.0808	0.0847	0.0860	0.0051
	OH	25	9,838	4,374	44.5	534	5.4	0.1040	0.1096	0.1067	−0.0046
	KK	21	9,788	2,963	30.3	791	8.1	0.0783	0.0892	0.0809	−0.0181
Vent	OT	63	9,838	6,554	66.6	2,886	29.3	0.1113	0.1107	0.1123	0.0155

Notes.—JR, Jiaolong Ridge seep genetic group; OH, Off Hatsushima seep genetic group; KK, Kuroshima Knoll seep genetic group; OT, Okinawa Trough vent genetic group; N, number of individuals; Variant, number of variant nucleotide sites; Poly, number of polymorphic nucleotide sites; Poly (%), percentage of polymorphic sites in variant sites; Private, number of unique SNPs; Private (%), percentage of unique SNPs in variant sites; *H*_exp_, expected heterozygosity under Hardy–Weinberg equilibrium; *H*_obs_, observed heterozygosity; *π*, nucleotide diversity; *F*_IS_, inbreeding coefficient. Two individuals from the Hatoma Knoll vent and three individuals from the Izena Hole Field vent in the Okinawa Trough showing high genetic similarities to those from the Off Hatsushima seep, inferred to be migrants from the Off Hatsushima seep, were excluded from the Okinawa Trough vent genetic group whereas included in the Off Hatsushima seep genetic group for genetic statistics estimation. Two individuals from the Izena Hole Field vent exhibiting genetic architecture of both the Okinawa Trough vent and the Off Hatsushima seep genetic groups, inferred to be hybrid descendants between individuals from these two genetic groups, were excluded from the Okinawa Trough vent genetic group for genetic statistics estimation. *H*_exp_, *H*_obs_, *π*, and *F*_IS_ were the average values of all variant nucleotide sites for each genetic group with details included in supplementary table S5, Supplementary Material online.

### Migration Dynamics

**Fig. 2.**
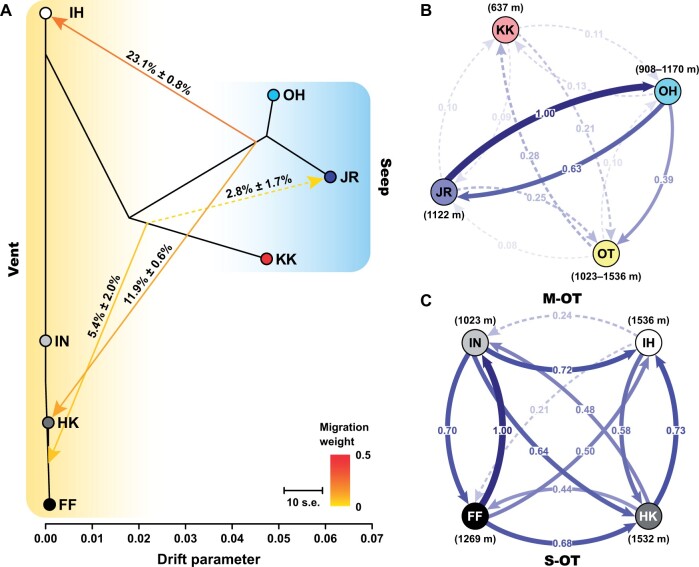
Migration dynamics of *Bathyacmaea nipponica*. (*A*) Four migration events (*m* = 4) amongst local populations detected by TreeMix. Arrows indicate the direction of migration, which are colored according to the migration weight. Direction of each arrow shows that of the migration event, and number on the arrow represents the jackknife estimate of the migration weight ± standard error. Dashed line indicates the migration event with a *P* value > 0.05. (*B*) Directional relative migration between pairs of genetic groups evaluated using divMigrate. (*C*) Directional relative migration between pairs of the vent local populations in the Okinawa Trough evaluated using divMigrate. In (*B*) and (*C*), arrows refer to the direction of gene flow, and numbers on the arrows represent the relative migration coefficients derived from the *G*_ST_ statistics. Stronger gene flows are represented by larger numbers, as well as the thicker and the darker colored lines. Dashshed lines represent limited gene flow with a coefficient < 0.3. Abbreviations for localities of local populations or genetic groups: JR, Jiaolong Ridge seep; FF, Futagoyama Field vent; HK, Hatoma Knoll vent; KK, Kuroshima Knoll seep; IN, Iheya North Field vent; IH, Izena Hole Field vent; OH, Off Hatsushima seep; S-OT, southern Okinawa Trough; M-OT, middle Okinawa Trough.

TreeMix analysis uncovered four migration events (*m* = 4; fig. 2*A*). Amongst them, three seep-to-vent migration events were statistically significant, including two from the phylogenetical ancestry of the Jiaolong Ridge and the Off Hatsushima seep populations to the Hatoma Knoll vent population in the southern Okinawa Trough (*P *<* *0.00001) and to the Izena Hole Field vent population in the middle Okinawa Trough (*P *<* *0.00001), as well as one from the Kuroshima Knoll seep population to the phylogenetical ancestry of the southern Okinawa Trough vent populations (*P *<* *0.005).

divMigrate analysis unveiled intense gene flow between the Jiaolong Ridge seep genetic group and the Off Hatsushima seep genetic group, weak gene flow from the Off Hatsushima seep genetic group to the Okinawa Trough vent genetic group, and very limited gene flow between the Kuroshima Knoll seep genetic group and the other two seep genetic groups in the Jiaolong Ridge and the Off Hatsushima (fig. 2*B*). Extensive gene flow was also observed between the vent local population pairs within the Okinawa Trough, with an overall stronger gene flow from the shallower to the deeper vent fields, especially for the vent local populations within the same region (i.e., within either the southern or the middle Okinawa Trough; fig. 2*C*).

### Demographic History

**Fig. 3.**
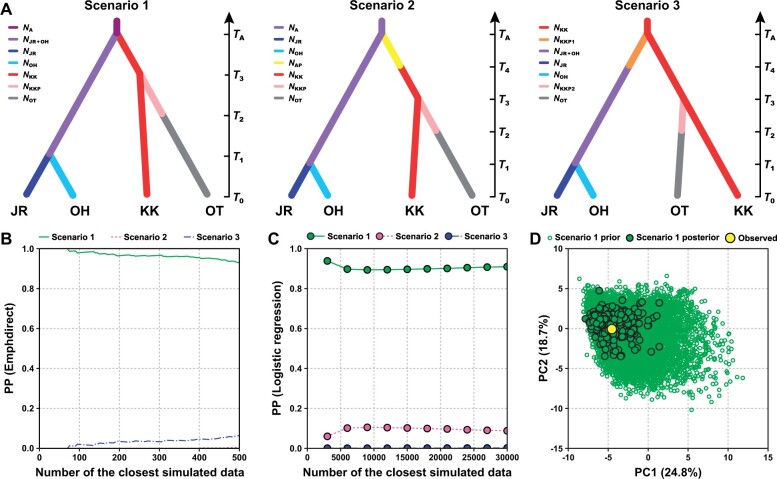
Demographic history of *Bathyacmaea nipponica* inferred from DIYABC. (*A*) Three demographic scenarios. The divergence times are not to scale (*T*_1_ < *T*_2_ < *T*_3_ < *T*_4_ < *T*_A_). *T*_0_ indicates present. (*B*) Posterior probabilities (PP) of each scenario estimated using the emphdirect method. (*C*) PP of each scenario calculated using the logistic regression method. (*D*) PCA showing the model fitness by comparing the observed data set with the data sets simulated from both the prior and posterior distribution of demographic parameters. Abbreviations: A, ancestral; AP, a few limpet larvae from the ancestral genetic group; JR, Jiaolong Ridge seep genetic group; OH, Off Hatsushima seep genetic group; KK, Kuroshima Knoll seep genetic group; KKP, a few limpet larvae from the Kuroshima Knoll seep genetic group; OT, Okinawa Trough vent genetic group.

Posterior probabilities evaluated based on the emphdirect and logistic regression methods consistently indicated that scenario 1 provided the most likely demographic history of *B*. *nipponica* (fig. 3*A*–*C* and supplementary table S6, Supplementary Material online). Moreover, the PCA plot of model checking showed that the observed data set was within the clusters of data sets simulated from both the prior and posterior distribution of demographic parameters (fig. 3*D*). This result implied that demographic parameters of scenario 1 estimated herein (table 2) fitted the data well (Cornuet et al. 2014).

**Table 2.** Demographic Parameters of Scenario 1 Estimated by DIYABC.

Demographic Parameters		Median	Mean	Quantile	
				5%	95%
Time scale in generations	*T*_1_	1,220	1,370	1,040	2,010
	*T*_2_	6,410	7,480	5,400	13,100
	*T*_3_	11,800	12,000	10,700	13,800
	*T*_A_	24,400	25,800	21,700	31,900
Effective population size	*N*_A_	97,400	95,600	87,200	99,200
	*N*_JR+__*O*__H_	38,900	41,300	7,410	84,500
	*N*_JR_	10,700	13,500	3,600	33,400
	*N*_OH_	51,400	52,300	25,700	84,800
	*N*_KK_	43,800	44,000	22,700	66,900
	*N*_KKP_	650	615	155	967
	*N*_OT_	98,200	97,800	94,900	99,600

Notes.—A, ancestral; JR, Jiaolong Ridge seep genetic group; OH, Off Hatsushima seep genetic group; KK, Kuroshima Knoll seep genetic group; KKP, a few limpet larvae from the Kuroshima Knoll seep genetic group; OT, Okinawa Trough vent genetic group. Two individuals from the Hatoma Knoll vent and three individuals from the Izena Hole Field vent in the Okinawa Trough were inferred to be immigrants from the Off Hatsushima seep, and two individuals from the Izena Hole Field vent exhibiting genetic architecture of both the Okinawa Trough vent and the Off Hatsushima seep genetic groups were inferred to be hybrid descendants between individuals from these two genetic groups. Therefore, these individuals were excluded from the demographic analysis to avoid potential bias.

Under this demographic scenario, genetic divergence between the deeper seep (i.e., Jiaolong Ridge and Off Hatsushima) genetic group and the shallower seep (i.e., Kuroshima Knoll) genetic group occurred ∼24,400 generations ago; opportunistic invasion by a small number of limpet larvae (∼650) from the Kuroshima Knoll seep genetic group to vent fields in the Okinawa Trough occurred ∼11,800 generations ago; establishment of the Okinawa Trough vent genetic group occurred ∼6,410 generations ago, and further genetic separation of the deeper seep genetic group into the Jiaolong Ridge and the Off Hatsushima seep genetic groups occurred ∼1,220 generations ago (table 2).

Amongst the four genetic groups, the Jiaolong Ridge seep genetic group had the smallest effective population size of ∼10,700, whereas the Okinawa Trough vent genetic group had the largest effective population size of ∼98,200 (table 2).

### Physical Ocean Modeling

**Fig. 4.**
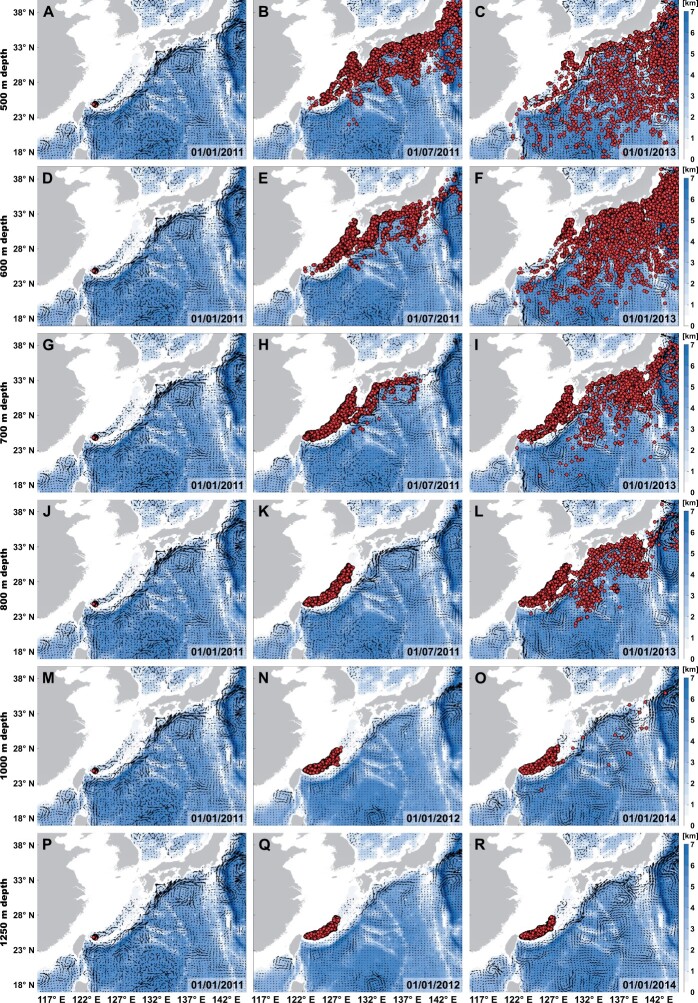
Representative distribution patterns of the numerical particles released from the Futagoyama Field vent coordinates. Particles were released at (*A*–*C*) 500, (*D*–*F*) 600, (*G*–*I*) 700, (*J*–*L*) 800, (*M*–*O*) 1,000, and (*P*–*R*) 1,250 m depths on January 1, 2011. The date shown in each submap is in the format of day/month/year. Color coding represents the total ocean depth (unit: km). Animations showing the entire series of experiments are available as supplementary movies 1–6 deposited in figshare.

Seasonal-mean lateral ocean currents in the study region at selected depths during the five model years (2011–2015) are illustrated in supplementary figure S1, Supplementary Material online. The time-mean lateral velocity along (or within) the Okinawa Trough decayed rapidly in the vertical direction, from ∼1 m/s at the sea surface to ∼0.1 m/s below 1,000 m depth, regardless of the seasons considered. The vertical decline of the lateral flow was also reflected in the numerical particle release experiments based on the daily transient velocity shown in figure 4*A*–*R* and supplementary movies 1–6: Dispersal of the numerical particles released from the Futagoyama Field vent coordinates in the Okinawa Trough became increasingly slower toward the greater depths. In addition, export of numerical particles from the Okinawa Trough into the open Northwest Pacific was largely constrained by the topographic features of the Okinawa Trough, particularly at and below 800 m depth (fig. 4*J*–*R* and supplementary movies 4–6). Notably, the Okinawa Trough appeared to be completely sealed at 1,250 m depth, as no numerical particles released from the Futagoyama Field vent coordinates traveled out of the Okinawa Trough throughout the entire experiment (fig. 4*P*–*R* and supplementary movie 6).

**Fig. 5.**
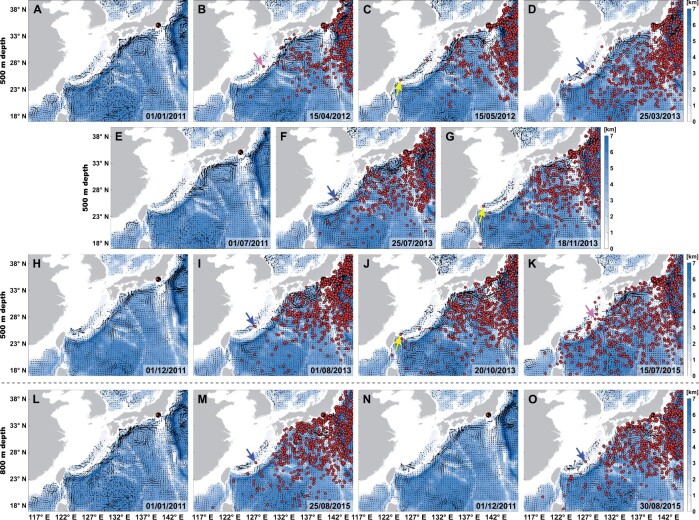
Representative distribution patterns of the numerical particles released from the Off Hatsushima seep coordinates. Particles released at 500 m depth on three dates in 2011, including on (*A*–*D*) 1 January, (*E*–*G*) 1 July, and (*H*–*K*) 1 December. Particles released at 800 m depth on two dates in 2011, including on (*L*, *M*) 1 January and (*N*, *O*) 1 December. Arrows indicate numerical particles entering the Okinawa Trough via different routes: magenta, via the water lanes east of the Okinawa Island; yellow, via the channel east of Taiwan; dark blue, via the Kerama Gap. The date shown in each submap is in the format of day/month/year. Color coding represents the total ocean depth (unit: km). Animations showing the entire series of experiments are available as supplementary movies 7–12 deposited in figshare.

The depth-dependent dispersal patterns of numerical particles released from the Off Hatsushima seep coordinates are illustrated in figure 5 and supplementary movies 7–12. In particular, a small number of numerical particles released at 500 m depth (fig. 5*A*, *E*, and *H*) invaded the Okinawa Trough via the water lanes east of the Okinawa Island in two out of three experiments launched in 2011, including one on 1 January (fig. 5*B* and supplementary movie 7) and the other on 1 December (fig. 5*K* and supplementary movie 9). Moreover, some numerical particles entered the Okinawa Trough through the channel east of Taiwan (fig. 5*C*, *G*, and *J*) and the Kerama Gap (fig. 5*D*, *F*, and *I*) in all three experiments launched in 2011 (supplementary movies 7–9). By contrast, water transport from the Off Hatsushima seep coordinates into the Okinawa Trough was topographically constrained at 800 m depth, because only one numerical particle entered the Okinawa Trough via the Kerama Gap in the experiment began either on 1 January (fig. 5*L* and *M* and supplementary movie 10) or 1 December 2011 (fig. 5*N and O* and supplementary movie 12). Furthermore, no numerical particles released at 800 m depth from the Off Hatsushima seep coordinates on July 1, 2011 were found to drift into the Okinawa Trough (supplementary movie 11). In all cases, numerical particles released from the Off Hatsushima seep coordinates were carried by the unsteady subtropical gyre recirculation and mesoscale ocean currents into the open Northwest Pacific before reaching the vicinity of the Okinawa Trough.

## Discussion

In the present study, we examined the demographic history and population connectivity of the deep-sea patellogastropod limpet *B*. *nipponica* by using an integrated population genomic and physical ocean modeling approach. Our results illustrated how the vent and seep populations of *B*. *nipponica* have diversified and connected under the complex interactions amongst local habitats, seafloor topography, and ocean currents.

The seven studied populations of *B*. *nipponica* formed four genetic groups that were primarily differentiated by habitat types, including three seep (i.e., Jiaolong Ridge, Off Hatsushima, and Kuroshima Knoll) and one vent (i.e., Okinawa Trough) genetic groups. These results were contrasted with those of a previous study based only on the nuclear *histone H3* and the mitochondrial *cytochrome c oxidase subunit I* (*cox1*) genes, in which *B*. *nipponica* in the Northwest Pacific was inferred to form one panmictic metapopulation (Chen et al. 2019). Amongst the three seep genetic groups detected herein, pairwise *F*_ST_ values, phylogenetic network, ADMIXTURE results, and PCA plots all revealed a pattern of genetic divergence that was not correlated with geographic distance (fig. 1*B*–*D* and *F* and supplementary table S4, Supplementary Material online). Particularly, genetic similarities between the Jiaolong Ridge and the Off Hatsushima seep genetic groups, which are ∼2,400 km apart, were remarkably higher than between these and the Kuroshima Knoll seep genetic group, which is located in the middle of the Jiaolong Ridge and the Off Hatsushima seeps. Nevertheless, the Jiaolong Ridge (depth: 1,122 m) and the Off Hatsushima seeps (depth: 908 to 1,170 m) are distributed in relatively deeper water compared with the Kuroshima Knoll seep (depth: 637 m), indicating that water depths might have played a role during the formation of these seep genetic groups. This inference was strongly supported by the results of DIYABC analysis (fig. 3, table 2, and supplementary table S6, Supplementary Material online), which uncovered that the ancestral population of *B*. *nipponica* may have initially diversified into two seep genetic groups ∼24,400 generations ago, with one distributed in the deeper seeps (i.e., Jiaolong Ridge seep and Off Hatsushima seep) and the other in the shallower seep (i.e., Kuroshima Knoll seep). Water depths have been noted as a key factor driving the diversification of animal groups in deep-sea chemosynthesis-based ecosystems, such as some vesicomyid clams in the Northwest Pacific (Fujikura et al. 2000). Nevertheless, we cannot rule out the possibility that other factors (e.g., local currents and geofluid chemistry) may have contributed to this as well.

Located in the fore-arc regions, the Off Hatsushima seep in the Sagami Bay and the Kuroshima Knoll seep in the Ryukyu Arc exhibit a high level of fluid seepage from the seafloor (Masuzawa et al. 1992; Takeuchi et al. 2007). In comparison, the Jiaolong Ridge seep in the South China Sea has displayed a weak supply of methane over the last two thousand years (ka) (Feng and Chen 2015). The relatively small pairwise *F*_ST_ values (fig. 1*B* and supplementary table S4, Supplementary Material online), high genetic similarities (fig. 1*C*, *D*, and *F*), and extensive gene flow (fig. 2*B*) between the Jiaolong Ridge and the Off Hatsushima seep genetic groups were in accordance with the results of DIYABC analysis, which revealed a very recent divergence (∼1,220 generations ago) of these two deeper seep genetic groups (table 2). The accurate generation time of *Bathyacmaea* remains unknown. With the assumption that *B*. *nipponica* has a generation time of one to two years (previous live-rearing experiments in aquaria under atmospheric pressure showed that adults of *B*. *nipponica* can live for at least one year by feeding on naturally occurring bacterial film) (Chen et al. 2019), genetic separation between the Jiaolong Ridge and the Off Hatsushima seep genetic groups would have occurred in a period (∼2.4 to 1.2 ka) when the Jiaolong Ridge seep underwent a reduced methane supply (Feng and Chen 2015). Such diminished geofluid released from the Jiaolong Ridge seep resulted in the vanishing of chemosynthetic communities on the ridge flanks (Feng and Chen 2015), which may also be responsible for the small effective population size of *B*. *nipponica* inhabiting there (table 2). In addition, the Luzon Strait between the marginal South China Sea and the open Northwest Pacific might have served as a physical barrier to promote such a recent genetic separation of *B*. *nipponica* living in the Jiaolong Ridge and the Off Hatsushima seeps, as has been inferred from other macrobenthos that colonize both sides of the Luzon Strait (e.g., Xu et al. 2018; Xiao et al. 2020).

Pairwise *F*_ST_ estimation, phylogenetic network, ADMIXTURE results (*K *=* *2), and PCA plots consistently implied that the Kuroshima Knoll seep genetic group had high genetic similarities to the vent genetic group established in the Okinawa Trough (fig. 1*B*–*D* and *F* and supplementary table S4, Supplementary Material online). The seep-to-vent migration event detected from the Kuroshima Knoll seep to the Okinawa Trough vents (fig. 2*A*) and the geographical proximity of these two regions further indicated that the Okinawa Trough vent genetic group may have been established following the historical invasion of limpet larvae from the Kuroshima Knoll seep. This inference was also supported by the results of DIYABC analysis, which unveiled that a small number of limpet larvae (∼650) from the Kuroshima Knoll seep might have invaded the Okinawa Trough ∼11,800 generations ago, adapted to the local environments, and thereafter formed the Okinawa Trough vent genetic group ∼6,410 generations ago (table 2). Assuming a generation time of one to two years for *B*. *nipponica* is appropriate (Chen et al. 2019), these patterns of population divergence would fit in a time window (i.e., larval invasion from the Kuroshima Knoll seep to the Okinawa Trough: ∼23.6 to 11.8 ka; population establishment in the Okinawa Trough vents: ∼12.8 to 6.4 ka) corresponding to the possible historical variation in strength and main course of the Kuroshio Current. Previous studies suggested that potentially due to the decrease of sea level and/or emergence of a Ryukyu-Taiwan land bridge, the Kuroshio mainstream had shifted toward the east of the Ryukyu Arc during the last glacial maximum and then swung back to the Okinawa Trough ∼14 to 7 ka (Jian et al. 2000; Ujiié et al. 2003; Lim et al. 2017). Such a shifting of the Kuroshio Current may have thus facilitated the historical migration of *B*. *nipponica* larvae from the Kuroshima Knoll seep into the Okinawa Trough, and afterwards accounted for their successful population expansion and local adaptation in vent fields within the trough. Nevertheless, it should be noted that our estimation of the demographic events suffered from an uncertainty of the limpet’s generation time. Determining its accurate generation time via laboratory larval culture experiments, although challenging for deep-sea organisms, is desired in the future to verify the inferences raised here.

Population genetic analyses of *F*_ST_ estimation, phylogenetic network reconstruction, ADMIXUTRE (*K *=* *3 and *K *=* *4), and PCA concordantly revealed that all the vent local populations in the Okinawa Trough belonged to one metapopulation (fig. 1*B*–*D* and *F* and supplementary table S4, Supplementary Material online), with stronger contemporary gene flow detected from the shallower to the deeper vent fields (fig. 2*C*). Physical ocean modeling uncovered a significant decline in the time-mean lateral velocity over depths within the Okinawa Trough (supplementary fig. S1, Supplementary Material online). Moreover, numerical particle release experiments demonstrated that water transport between the Okinawa Trough and the open Northwest Pacific was topographically constrained, especially at and below 800 m depth (fig. 4*J*–*R* and supplementary movies 4–6). These different lines of evidence implied that the larvae of *B*. *nipponica* may mainly disperse along the intermediate to the deeper ocean currents, which is similar to the larvae of *S*. *crosnieri* (Miyake et al. 2010; Xiao et al. 2020). Therefore, the overall concave topography of the Okinawa Trough may have trapped the dispersal of *B*. *nipponica* larvae (Mitarai et al. 2016), and afterwards led to a high level of genetic homogeneity (e.g., small pairwise *F*_ST_ values between the vent local population pairs), a high degree of inbreeding (i.e., the highest *F*_IS_ value amongst the four genetic groups), and extensive contemporary gene flow with a direction from the shallower to the deeper vents for individuals inhabiting the Okinawa Trough. Such a larval dispersal potential of *B*. *nipponica* would also help to explain its different population segregation patterns in comparison with those of the bathymodioline mussel *G*. *platifrons*. This mussel has been considered to produce planktotrophic larvae that may mainly disperse in the upper currents, with the local populations in vent fields in the Okinawa Trough and the Off Hatsushima seep in the Sagami Bay sharing high genetic similarities (Xu et al. 2018). Moreover, the largest number of private SNPs identified in the Okinawa Trough vent genetic group implied the adaptive capabilities of *B*. *nipponica* to the local vent environments, which may have also contributed to the highest heterozygosity of this genetic group compared with all the other genetic groups.

In addition, occurrence of weak contemporary gene flow from the Off Hatsushima seep to the Okinawa Trough vent genetic groups of *B*. *nipponica* was accordantly unveiled by the phylogenetic network, ADMIXTURE results (*K *=* *4), and PCA plots; that is, five Okinawa Trough vent individuals inferred to be immigrants from the Off Hatsushima seep, and two Okinawa Trough vent individuals inferred to be hybrid descendants between individuals from the Okinawa Trough vent and the Off Hatsushima seep genetic groups (fig. 1*C*, *D*, and *F*). Consistent with the results of these population genomic analyses, physical ocean modeling deciphered the intrusion of some water from the Off Hatsushima seep coordinates into the Okinawa Trough at 500 and 800 m depths mainly via the channel east of Taiwan and the Kerama Gap, although such invasion was largely restricted at 800 m depth due to the topographic constraints of the Okinawa Trough (fig. 5 and supplementary movies 7–12). This water body belongs to the North Pacific Intermediate Water (Bostock et al. 2010; Nakamura et al. 2013). Hence, transport of the North Pacific Intermediate Water might have facilitated the contemporary dispersal of a few *B*. *nipponica* larvae from the Off Hatsushima seep into the Okinawa Trough. These larvae then settled in different vent fields in the Okinawa Trough, and subsequently resulted in hybridization between individuals belonging to the Off Hatsushima seep and the Okinawa Trough vent genetic groups.

At present, deep-sea vent and seep ecosystems are facing an increasing threat from ongoing or upcoming anthropogenic disturbances, most noticeably natural resources exploitation (e.g., minerals and ores in vents and methane hydrates, oil, and gas in seeps) (Ramirez-Llodra et al. 2011; Levin et al. 2020). These disturbances have been predicted to cause adverse effects on the entire marine ecosystems, leading to degradation and loss of habitats, reduction in biodiversity, and alteration of community structure and ecological function (Ramirez-Llodra et al. 2011; Levin et al. 2020). Extraction of deep-sea ores has already been tested in hydrothermal vents in the Okinawa Trough (Okamoto et al. 2019), making this area potentially one of the world’s first deep-sea mining sites in the near future. Therefore, the results generated herein would have practical implications for the conservation of vent- and seep-associated organisms, especially for those exhibiting similar dispersal potential as *B*. *nipponica* (Mills et al. 2009): Although the same species dwells in both vents and seeps, existence of habitat-linked genetic structure implies that quick recolonization from one habitat to another cannot be assumed. As a consequence, individual sites within the same habitat and/or geographic region, such as some vent fields in the Okinawa Trough, should be identified and assigned as protected areas to ensure a high resilience is maintained within the local population groups. Furthermore, recent drilling campaigns have deepened our understanding of the distribution and storage of gas hydrate in seep ecosystems in the South China Sea, at the same time, drawn our attention to monitor and protect the chemosynthesis-based communities in this region (Feng et al. 2018). Detectable genetic divergence between the South China Sea and the Northwest Pacific populations underlines the barrier effect of the Luzon Strait on larval dispersal of *B*. *nipponica*, as has been demonstrated in other deep-sea organisms inhabiting these two areas (e.g., Xu et al. 2018; Xiao et al. 2020). Due to the marginal geographic feature of the South China Sea and the genetic distinctness of its seep communities, we therefore suggest that seep ecosystems in the South China Sea should also be given a high priority of biodiversity conservation when designating deep-sea reserves and marine protected areas in the global ocean (Baco et al. 2016; Levin et al. 2020).

## Conclusions

Using an integrated approach combining population genomics and physical ocean modeling, four distinct genetic groups with a habitat-linked pattern were disclosed for the deep-sea patellogastropod limpet *B*. *nipponica* in the Northwest Pacific, including three seep genetic groups inhabiting hydrocarbon seeps in the Jiaolong Ridge, the Kuroshima Knoll, and the Off Hatsushima, as well as one vent genetic group dwelling in four hydrothermal vents in the Okinawa Trough. The assumed lecithotrophic larvae of *B*. *nipponica* were considered to mainly disperse along the intermediate to the deeper ocean currents. The most likely demographic history of *B*. *nipponica* was deciphered to have occurred in the following sequences: 1) an initial split into two seep genetic groups, one deeper (i.e., Jiaolong Ridge and Off Hatsushima) and one shallower (i.e., Kuroshima Knoll); 2) a small number of limpet larvae from the shallower seep genetic group invaded vent fields in the Okinawa Trough, adapted to the local environments, and thereafter established a distinct vent genetic group; and 3) two deeper seep populations diverged very recently into two distinct genetic groups with detectable genetic differentiation. Assuming a generation time of one to two years for *B*. *nipponica*, formation of its vent genetic group may have been linked to the historical shifting of the Kuroshio Current. Additionally, diversification of the two deeper seep genetic groups might be associated with the recent reduction of seepage intensity in the Jiaolong Ridge seep along with the barrier effect of the Luzon Strait. Physical ocean modeling results elucidated the seafloor topography of the Okinawa Trough may have contributed to the genetic homogeneity of *B*. *nipponica* among different vent local populations in this region, and the North Pacific Intermediate Water might have facilitated the opportunistic migration of a few limpet larvae from the Off Hatsushima seep in the Sagami Bay into the Okinawa Trough, and thus led to their hybridization in the Okinawa Trough vents. Altogether, this study has enhanced our knowledge on the historical population divergence and contemporary gene flow of deep-sea organisms inhabiting hydrothermal vents and hydrocarbon seeps under the intricate interactions amongst local habitats, seafloor topography, and ocean currents. More importantly, these results could serve as the scientific basis for a better conservation of chemosynthesis-based ecosystems and an effective establishment of marine protected areas.

## Materials and Methods

### Sample Collection and Genomic DNA Extraction

A total of 123 individuals of *B*. *nipponica* were collected from four hydrothermal vents and three hydrocarbon seeps in the Northwest Pacific between 2002 and 2016 (fig. 1*A* and supplementary table S7, Supplementary Material online). The four vent fields included the Futagoyama Field (Waka Site) and the Hatoma Knoll in the southern Okinawa Trough, and the Iheya North Field (Iheya North Original Site) and the Izena Hole Field (Jade Site) in the middle Okinawa Trough (Nakajima et al. 2014). The three seep areas included the Jiaolong Ridge (also known as Formosa Ridge or Site F) in the South China Sea (Zhao et al. 2020), the Kuroshima Knoll in the southern Ryukyu Arc, and the Off Hatsushima in the Sagami Bay. Specimens were either frozen at −80 °C or preserved in 99.5% ethanol. Genomic DNA was extracted from the foot of each individual using the cetyltrimethylammonium bromide method (Stewart and Via 1993). Purity, integrity, and quality of DNA were examined using a NanoDrop ND-1000 spectrophotometer (Thermo Fisher Scientific, Wilmington, DE), 1.0% agarose gel electrophoresis, and a Qubit 2.0 Fluorometer (Life Technologies, Carlsbad, CA), respectively.

### Genome Survey Sequencing and Assembly

To provide a reference genome for SNP identification, genome survey sequencing was performed for an individual of *B*. *nipponica* (i.e., JR-5) in Novogene (Beijing, China). A DNA sequencing library with an insert size of 350 bp was constructed using the NEBNext DNA Library Prep Kit and paired-end sequenced on an Illumina NovaSeq 6000 sequencer with a read length of 150 bp. Raw reads were filtered using Trimmomatic v.0.38 (Bolger et al. 2014) to remove adapters and low-quality reads with the following settings: ILLUMINACLIP: Adapters.fas: 2:30:10, LEADING = 10, TRAILING = 10, SLIDINGWINDOW = 4:15, and MINLEN = 40. Obtained high-quality reads were assembled using Platanus v.1.2.4 (Kajitani et al. 2014) under the default parameters. Assembly statistics were evaluated using the Perl script assemblathon_stats.pl (Bradnam et al. 2013), and assembly completeness was accessed using BUSCO v.3.0.2 (Simão et al. 2015) based on the metazoa_odb9 database.

### Genotyping by Sequencing and Genome-Wide SNP Identification

Genotyping by sequencing was performed in Novogene (Beijing, China). In brief, genomic DNA from each individual was digested using the restriction enzymes *MseI* and *MspI*. Pooled DNA libraries with insert sizes ranging from 240 to 265 bp were paired-end sequenced on an Illumina NovaSeq 6000 sequencer with a read length of 144 bp. Raw reads of each individual were filtered to remove adapters and poor-quality reads using fastp v.0.20.0 (Chen et al. 2018) under the following settings: --detect_adapter_for_pe and --trim_poly_x, set --qualified_quality_phred to 15, --unqualified_percent_limit to 30, --n_base_limit to 0, and set --max_len1, --max_len2, and --length_required all to 130. Obtained high-quality reads of each individual were mapped to the survey genome of *B*. *nipponica* using the MEM algorithm implemented in BWA v.0.7.17 (Li and Durbin 2009). The resultant .sam files were individually converted to .bam files and sorted using SAMtools v.1.9 (Li et al. 2009). Duplicate products of polymerase chain reaction and reads mapped to different positions of the survey genome were removed using Sambamba v.0.7.1 (Tarasov et al. 2015). Calling of SNPs was performed under a combination of the mpileup (-q 10 -Q 15 --skip-indels) and the call options (--multiallelic-caller --variants-only) implemented in BCFtools v.1.9 (Li 2011). Filtration was conducted using VCFtools v.0.1.16 (Danecek et al. 2011) to retain SNPs that meet the following criteria: 1) with two alleles (--min-alleles 2 --max-alleles 2); 2) with a sequencing coverage depth between 5 and 200 (--minDP 5 --maxDP 200); 3) with a genotype quality ≥ 20 (--minGQ 20); 4) presence in ≥ 80% of all individuals (--max-missing 0.8); 5) with a global minor allele frequency ≥ 0.02 (--maf 0.02); 6) SNP with an observed heterozygosity (*H*_obs_) < 0.5 calculated using popStats (Skoglund et al. 2015) to avoid inclusion of potential paralogues (Hohenlohe et al. 2011). Based on the statistics of SNP per genomic locus retained after the sixth filtering criterion, all SNPs located in genomic locus with more than nine SNPs were further discarded to reduce potential sequencing or genotyping errors. Formatting of the resultant final SNP data set for downstream analyses was achieved using PGDSpider v.2.1.1.5 (Lischer and Excoffier 2012) and Stacks v.2.5 (Rochette et al. 2019).

### Population Divergence Analyses and Genetic Statistics Estimation

Genetic differentiation between the local population pairs represented by pairwise *F*_ST_ was estimated using Arlequin v.3.5.2.2 (Excoffier and Lischer 2010), with 10,000 permutations performed to test for significance. A phylogenetic network was constructed with the NeighborNet method implemented in SplitsTree v.4.15.1 (Huson 1998), based on the normalized distances estimated with the uncorrectedP algorithm and the option of Handle Ambiguous States set to MatchStates. Population genetic structure was investigated using two methods with only one SNP per locus retained to reduce potential bias derived from linkage disequilibrium. A maximum likelihood (ML) estimation method implemented in ADMIXTURE v.1.3.0 (Alexander et al. 2009) was applied to examine population structure and individual ancestry, with the number of genetic groups represented by *K* predefined from one to seven. The number of genetic groups that best fit the data (i.e., the optimal *K*) was assessed using the cross-validation (CV) procedure implemented in ADMIXTURE with the --cv flag. The barplot function in R was used for result visualization with the selected *K*. The PCA implemented in the R package SNPRelate (Zheng et al. 2012) was applied to further investigate genetic divergence amongst all individuals. Several population genetic statistics, including expected heterozygosity (*H*_exp_), observed heterozygosity (*H*_obs_), nucleotide diversity (*π*), and inbreeding coefficient (*F*_IS_), were calculated using the POPULATIONS module implemented in Stacks v.2.5 (Rochette et al. 2019).

### Migration Dynamics Analyses

Migration events amongst local populations were evaluated using TreeMix v.1.13 (Pickrell and Pritchard 2012). Specifically, an ML tree was constructed based on the SNP data set (only one SNP per locus retained) with migration events added to this tree one at a time to infer migration event models along with the -global plus -se options. Besides, migration patterns between pairs of genetic groups and pairs of the selected local populations were examined using divMigrate-online (Sundqvist et al. 2016) based on the *G*_ST_ statistic as a measure of genetic differentiation.

### Demographic History Reconstruction

Demographic history was reconstructed using the approximate Bayesian computation (ABC) analysis implemented in DIYABC v.2.1.0 (Cornuet et al. 2014). According to the results of pairwise *F*_ST_ estimation, phylogenetic network, population structure, and migration dynamics (details refer to the Results section), three demographic scenarios were hypothesized to infer how the four identified genetic groups of *B*. *nipponica* had formed and diversified in the Northwest Pacific (fig. 3*A*). Each scenario was predefined by a number of demographic parameters expressed as the number of generations back in time (i.e., *T*_1_ < *T*_2_ < *T*_3_ < *T*_4_ < *T*_A_) with details summarized in supplementary table S8, Supplementary Material online.

In scenario 1, *B*. *nipponica* initially diversified into two seep genetic groups at *T*_A_. The first seep genetic group inhabited the shallower seep area in the Kuroshima Knoll and formed the Kuroshima Knoll seep genetic group. A few limpet larvae from the Kuroshima Knoll seep genetic group (i.e., *N*_KKP_) invaded and settled in the Okinawa Trough vents by chance at *T*_3_, which later diverged to form the Okinawa Trough vent genetic group at *T*_2_. The second seep genetic group inhabited the deeper seep areas, which diverged into two distinct seep genetic groups in the Jiaolong Ridge and the Off Hatsushima seeps at *T*_1_. In scenario 2, *B*. *nipponica* inhabited the deeper seep areas was hypothesized to be ancestral. A few limpet larvae from this ancestral genetic group (i.e., *N*_AP_) incidentally invaded and settled in the shallower seep area in the Kuroshima Knoll at *T*_A_, which gradually formed the Kuroshima Knoll seep genetic group at *T*_4_. Afterward, a few limpet larvae from the Kuroshima Knoll seep genetic group (i.e., *N*_KKP_) occasionally dispersed and settled in the Okinawa Trough vents at *T*_3_, which thereafter formed the Okinawa Trough vent genetic group at *T*_2_. More recently, the ancestral deeper seep genetic group further diversified into two distinct seep genetic groups in the Jiaolong Ridge and the Off Hatsushima seeps at *T*_1_. In scenario 3, *B*. *nipponica* inhabited the shallower seep area in the Kuroshima Knoll was speculated to be ancestral. A few limpet larvae from the Kuroshima Knoll seep genetic group (i.e., *N*_KKP1_) fortuitously invaded the deeper seep areas at *T*_A_, which then established the deeper seep genetic group at *T*_4_. Furthermore, a few other limpet larvae from the Kuroshima Knoll seep genetic group (i.e., *N*_KKP2_) incidentally entered and settled in the Okinawa Trough vents at *T*_3_, which afterward formed the Okinawa Trough vent genetic group at *T*_2_. Lately, the deeper seep genetic group further differentiated to establish two distinct seep genetic groups in the Jiaolong Ridge and the Off Hatsushima seeps at *T*_1_.

To obtain robust results, a total of 3,000,000 simulations were performed as recommended by DIYABC for all three demographic scenarios. Summary statistics, including proportion of zero values, mean and variance of nonzero values, and mean of complete distribution, were calculated for each genetic group. In addition, summary statistics, including mean and variance of nonzero values of *F*_ST_, proportion of zero values and mean of complete distribution *Nei’*s distances, were calculated between pairs of genetic groups. Posterior probabilities and 95% confidence intervals for each scenario were estimated based on the 500 simulated data closest to the observed data using the emphdirect method and 1% of the simulated data closest to the observed data using the logistic regression method. Posterior distribution of each demographic parameter of the best scenario was calculated according to 1% simulated data closest to the observed data with the logistic regression method. Fitness of the parameter priors to the best scenario was illustrated using PCA with the function of Perform Model Checking.

### Physical Ocean Modeling

Population genomic analyses conducted in the present study uncovered extensive contemporary gene flow of *B*. *nipponica* between its vent local populations in the Okinawa Trough, and weak contemporary gene flow from its Off Hatsushima seep to the Okinawa Trough vent genetic groups (details refer to the Results section). To establish a deeper understanding of these gene flow patterns from an oceanographic aspect, physical ocean modeling data were further analysed to uncover the 1) seasonal-mean lateral ocean current patterns and 2) topographic constraints and fluid dynamics in the study region via two sets of numerical particle release experiments.

Velocity fields of lateral ocean currents were extracted from the HYCOM + NCODA Global 1/12° Reanalyses (experiment sequence: 53.X) modeling output (https://www.hycom.org, last accessed April 10, 2021) in the study region. This set of ocean modeling data has integrated multiple sources of ocean observational records, including satellite measurements of the sea surface height/temperature and in-situ hydrographic information documented by the XBTs/Argo floats/moored buoys. Seasonal-mean lateral ocean current patterns at 0, 500, 800, 1,000, and 1,250 m depths were elucidated by averaging the daily velocity fields within each season (spring: March–May; summer: June–August; fall: September–November; winter: December–February) across an ensemble of five model years (2011–2015). This calculation effectively filtered out high-frequency processes, such as transient mesoscale eddies, which allowed investigation of the present-day large-scale ocean circulations.

To understand the intensive contemporary gene flow between the vent local populations of *B*. *nipponica* within the Okinawa Trough (details refer to the Results section), the first set of numerical particle release experiments were performed by uniformly releasing numerical particles within a 25-km radius from the Futagoyama Field vent coordinates (i.e., the westernmost vent location in the Okinawa Trough included herein), which then flew passively with ocean currents for around three years since 2011. Based on the seasonal-mean lateral ocean current patterns in the study region and the migration dynamics of *B*. *nipponica* (details refer to the Results and Discussion sections), *B*. *nipponica* larvae were considered to mainly disperse in the intermediate to the deeper water similar to those of *S*. *crosnieri* (Miyake et al. 2010; Xiao et al. 2020). Therefore, a series of depths ranging from the intermediate to the deeper water were selected for numerical particle release experiments in the Okinawa Trough region to verify such an inference (supplementary table S9, Supplementary Material online).

Previous studies suggested that the North Pacific Intermediate Water can intrude into the Okinawa Trough mainly via the channel east of Taiwan and the Kerama Gap (Bostock et al. 2010; Nakamura et al. 2013), which may explain the weak contemporary gene flow of *B*. *nipponica* from the Off Hatsushima seep in the Sagami Bay to vent fields in the Okinawa Trough (details refer to the Results section). To examine this hypothesis, the second set of numerical particle release experiments were performed by uniformly releasing numerical particles within a 25-km radius from the Off Hatsushima seep coordinates at 500 and 800 m depths. These two depths were selected based on the seasonal-mean lateral ocean current patterns in the study region (details refer to the Results section), topographic constraints of the Okinawa Trough inferred from the results of the first set of numerical particle release experiments (details refer to the Results section), and depth range of the North Pacific Intermediate Water (Bostock et al. 2010; Nakamura et al. 2013). Numerical particle release experiments unveiled the locations for numerical particles released from the Off Hatsushima seep coordinates to invade the Okinawa Trough varied with the release dates. Therefore, experiments performed on three representative release dates spanning 2011 at two selected water depths till the end of 2015 are presented herein to illustrate the intrusion of the North Pacific Intermediate Water into the Okinawa Trough (supplementary table S9, Supplementary Material online).

The two sets of numerical particle release experiments were analyzed based on the horizontal and time-varying ocean current velocity. To attain statistically robust results, a broader domain (15°N–40°N × 115°E–180°E) than the study region was selected and over 10,000 numerical particles were released for each set of experiments. This approach accommodated long-range trajectories of particles following the Pacific Ocean currents (e.g., the Kuroshio Extension). Trajectories of these particles were calculated using a step-adapting fourth/fifth-order Runge–Kutta method with interpolations done using a cubit method (Wang et al. 2016). Seasonal-mean lateral ocean currents and distribution patterns of numerical particles were visualized using MATLAB v. R2018a (https://www.mathworks.com/products/matlab.html), aiming to reflect the topographic constraints and fluid dynamics of the study region. No data are available on the swimming capability of *B*. *nipponica* larvae. Therefore, the present study is not applicable for determining the exact travel time of *B*. *nipponica* larvae along ocean currents, such as from the Off Hatsushima seep in the Sagami Bay to vent fields in the Okinawa Trough.

## Supplementary Material

Supplementary data are available at *Molecular Biology and Evolution* online.

## Supplementary Material

msab278_Supplementary_Data
