# Supplementary material for: Hidden Historical Habitat-Linked Population Divergence and Contemporary Gene Flow of a Deep-Sea Patellogastropod Limpet
Source: Mol Biol Evol. 2021 Sep 17;38(12):5640–54. doi: 10.1093/molbev/msab278 (PMC8662656; doi:10.1093/molbev/msab278)
Supplement: msab278_Supplementary_Data [file msab278_supplementary_data.zip › Supplementary Figure.pdf]

Supplementary Figure

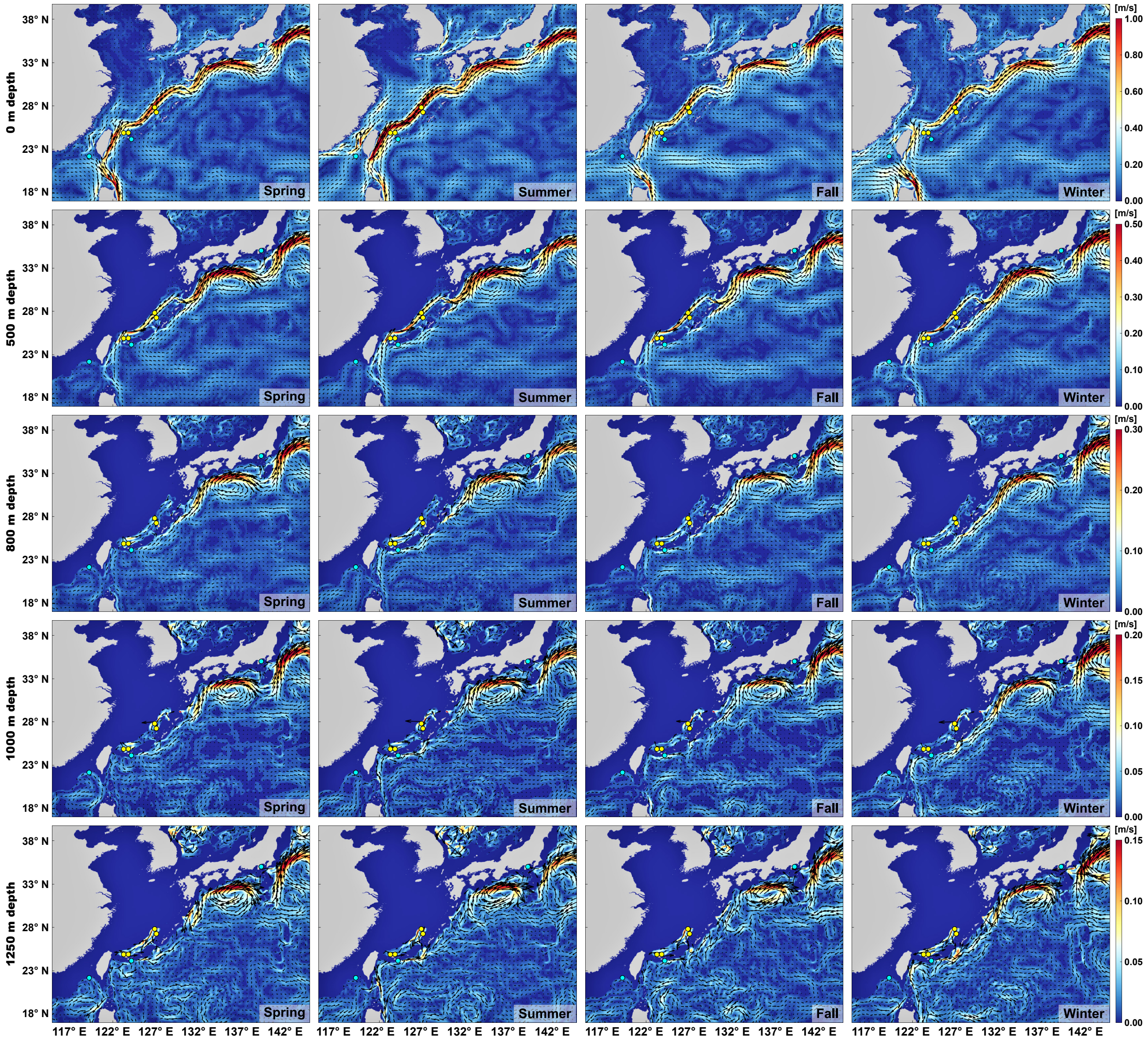

Supplementary Figure S1. Time-mean lateral velocity in the study region. The velocities are shown at 0, 500, 800, 1,000 and 1,250 m depths for spring (March–May), summer (June–August), fall (September–November) and winter (December–February) based on the HYCOM + NCODA Global 1/12° Reanalyses (experiment sequence: 53.X) modelling output for the five model years (2011–2015). Arrows indicate the directions and relative magnitudes of the lateral velocity vectors. Color coding indicates the absolute magnitude of the lateral velocity (unit: m/s). Vents and seeps are indicated by yellow and blue dots, respectively. Locality names refer to the legend of figure 1.
